# Improved isotopic model based on ^15^N tracing and Rayleigh‐type isotope fractionation for simulating differential sources of N_2_O emissions in a clay grassland soil

**DOI:** 10.1002/rcm.8374

**Published:** 2019-02-15

**Authors:** Antonio Castellano‐Hinojosa, Nadine Loick, Elizabeth Dixon, G. Peter Matthews, Dominika Lewicka‐Szczebak, Reinhard Well, Roland Bol, Alice Charteris, Laura Cardenas

**Affiliations:** ^1^ Department of Microbiology, Faculty of Pharmacy University of Granada. Campus Cartuja 18071 Granada Spain; ^2^ Rothamsted Research, North Wyke Okehampton EX20 2SB UK; ^3^ School of Geography, Earth and Environmental Sciences University of Plymouth Davy Building, Drake Circus Plymouth PL4 8AA UK; ^4^ Thünen Institute of Climate‐Smart Agriculture Bundesallee 65 38116 Braunschweig Germany; ^5^ Agrosphere (IBG‐3) Institute of Bio‐ and Geosciences Forschungszentrum Jülich 52428 Jülich Germany; ^6^ Department of Soil Microbiology and Symbiotic Systems Estación Experimental del Zaidín 18080 Granada Spain

## Abstract

**Rationale:**

Isotopic signatures of N_2_O can help distinguish between two sources (fertiliser N or endogenous soil N) of N_2_O emissions. The contribution of each source to N_2_O emissions after N‐application is difficult to determine. Here, isotopologue signatures of emitted N_2_O are used in an improved isotopic model based on Rayleigh‐type equations.

**Methods:**

The effects of a partial (33% of surface area, treatment 1c) or total (100% of surface area, treatment 3c) dispersal of N and C on gaseous emissions from denitrification were measured in a laboratory incubation system (DENIS) allowing simultaneous measurements of NO, N_2_O, N_2_ and CO_2_ over a 12‐day incubation period. To determine the source of N_2_O emissions those results were combined with both the isotope ratio mass spectrometry analysis of the isotopocules of emitted N_2_O and those from the ^15^N‐tracing technique.

**Results:**

The spatial dispersal of N and C significantly affected the quantity, but not the timing, of gas fluxes. Cumulative emissions are larger for treatment 3c than treatment 1c. The ^15^N‐enrichment analysis shows that initially ~70% of the emitted N_2_O derived from the applied amendment followed by a constant decrease. The decrease in contribution of the fertiliser N‐pool after an initial increase is sooner and larger for treatment 1c. The Rayleigh‐type model applied to N_2_O isotopocules data (δ^15^N^bulk^‐N_2_O values) shows poor agreement with the measurements for the original one‐pool model for treatment 1c; the two‐pool models gives better results when using a third‐order polynomial equation. In contrast, in treatment 3c little difference is observed between the two modelling approaches.

**Conclusions:**

The importance of N_2_O emissions from different N‐pools in soil for the interpretation of N_2_O isotopocules data was demonstrated using a Rayleigh‐type model. Earlier statements concerning exponential increase in native soil nitrate pool activity highlighted in previous studies should be replaced with a polynomial increase with dependency on both N‐pool sizes.

## 1 INTRODUCTION

Agricultural soils rely on external nitrogen (N) inputs and constitute a major source of nitrous oxide (N_2_O) and nitric oxide (NO) emissions, accounting for around 10% of greenhouse gas (GHG) emissions from human activities^1^ and contributing to the formation of acid rain, eutrophication and ground level ozone.^2^ In soil, nitrification and denitrification are the most important microbial processes involved in the production of N_2_O, requiring high and low oxygen (O_2_) concentrations for the activation of each process, respectively. Moreover, when denitrification occurs, N applied to soils can be emitted back to the atmosphere as dinitrogen (N_2_). Many observations have suggested that sequential synthesis of denitrification enzymes is responsible for the delay in N_2_ appearance relative to N_2_O.^3^, ^4^, ^5^

Amongst the strategies to identify N_2_O sources in the soil and their variation in space and time, the study of the natural abundance of stable isotopic signatures of N_2_O,^6^, ^7^ such as the δ^15^N and δ^18^O values and the ^15^N site preference (SP), have gained attention ever since the early 2000s.^8^, ^9^, ^10^ The N_2_O produced from denitrification in soils tends to be associated with δ^15^N signatures with values in the range of −13 to −54‰^11^, ^12^ while those derived from nitrification are up to −60‰.^11^, ^13^ Moreover, reduction of N_2_O to N_2_ from denitrifying bacteria can be determined by isotopic discrimination as a consequence of the difference in reaction rates of the isotopically light (^14^N, ^16^O) and heavy (^15^N, ^18^O) molecules of N_2_O.^14^, ^15^, ^16^ Interpretation of N_2_O isotopomers as indicators of source processes has also been developed.^17^, ^18^ This approach is based on the difference in ^15^N occupation of the peripheral (β) and central N‐positions (α) of the linear molecule that defines the intra‐molecular ^15^N SP.^19^, ^20^ The SP is not dependent on the isotopic signature of the precursor,^21^ in contrast to average δ^15^N and δ^18^O values of N_2_O. However, Sutka et al^22^ found that the SP is increased during fungal denitrification and nitrification whereas N_2_O reduction via denitrification increases the SP by increasing the α‐site ^15^N‐enrichment in the residual N_2_O.^9^, ^15^ Wu et al^23^ subsequently quantified the potential bias on SP‐based N_2_O source partitioning using a closed‐system model.

Nitrogen fertiliser application to agricultural land can affect the isotopic signature of N_2_O and result in two different pools of emissions: pool 1 from fertiliser addition and pool 2 from the native soil N. In addition to those two pools, spatial heterogeneity of denitrification can have a significant impact on N‐isotope patterns which might only occur in situations where available N and C are added at the same time, e.g. slurry, grazing excreta, urea fertiliser.^24^, ^25^, ^26^, ^27^ The isotope fractionation during N_2_O production^7^, ^12^ and reduction,^15^, ^16^ or when both processes take place simultaneously,^26^ has been previously reported. Moreover, a comprehensive review of isotope effects and isotope modelling approaches was recently presented by Denk et al.^28^ Previously, using a Rayleigh equation to describe isotopic fractionation,^29^ Well and Flessa^12^ concluded that the isotopic fingerprint of soil‐emitted N_2_O is a useful parameter to evaluate the contribution of different processes to the N_2_O flux in soils. However, the spatial extent and specific denitrification rates of hypothesized pools could only be constrained by fitting measured and modelled δ^15^N^bulk^ values, which were associated with considerable uncertainties on the volume and denitrification rates of the assumed pools. Modelling the isotope fractionation during production and reduction based on the measured temporal pattern of the δ^15^N^bulk^‐N_2_O values suggested that there was a multi‐pool (non‐homogenous) distribution of nitrate (NO_3_^−^) in the soil.^25^ Thus, evaluation of isotopologue signatures for identifying source processes was hampered by the simultaneous occurrence of several factors contributing to the time course of isotopic signatures, which could thus not be fully explained. In this sense, Lewicka‐Szczebak et al^26^ showed that higher denitrification rates resulted in decreasing net isotope effects during N_2_O production for ^15^N using a modelling approach. For N_2_O reduction, clearly diverse net isotope effects were observed for the two distinct soil pools. In addition, in a laboratory incubation carried out at different saturation levels for a grassland soil, Cardenas et al^30^ found that added N produced higher denitrification rates than soil N, resulting in less isotopic fractionation.

The kinetics of N transformations in soils has been previously explored using an isotopic model based on Rayleigh‐type equations.^26^ This model was developed to simulate δ^15^N values of N_2_O using process rates and associated fractionation factors, but assumptions had to be made for some of the model parameters due to a lack of available data. The model is able to evaluate the progress in nitrate consumption and the accompanying isotope effect by fitting the δ^15^N values for the produced N_2_O where the δ^15^N values of the residual N_2_O are calculated based on the known N_2_O reduction ratio. The latter ratio is calculated from direct measurements of the isotopic signature of the remaining unreduced N_2_O. The isotopic signature of the instantaneously produced N_2_O and the fraction of unreduced N_2_O are calculated, based on direct measurements of N_2_O and N_2_ fluxes. A more comprehensive description of the calculation methods and model construction can be found in Lewicka‐Szczebak et al.^26^ In this context, the aim of the present study was to parameterise the previous two‐pool model via determination of the N_2_O production and consumption as well as the N_2_O isotopocule signatures of emitted N_2_O in a soil treated with a partial and total dispersal of added N and C. The N_2_O isotopocule data were used to determine the importance of N_2_O emission from different pools using a Rayleigh‐type model. Controlling the soil volume of pool 1 we assessed the specific denitrification rates of pools 1 and 2 and independently evaluated the contribution of each pool to the total N_2_O flux using a parallel ^15^N‐tracing experiment. By applying isotopically labelled N, we were able to gain a deeper insight into the proportion of added N that produced the emitted N_2_O to estimate the magnitude of pool‐derived fluxes.

## 2 EXPERIMENTAL

### 2.1 Set up

A clayey pelostagnogley soil of the Hallsworth series (pH in water, 5.6; total N, 0.5%; ammonium N, 6.1 mg kg^−1^ dry soil; total oxidized N, 15.1 mg kg^−1^ dry soil; organic matter, 11.7%; clay, 44%; silt, 40%; sand, 15%; w/w) was collected in November 2013 from a typical grassland in SW England, located at Rothamsted Research, North Wyke, UK (50° 46′ 50″ N, 3° 55′ 8″ W). Spade‐squares (20 × 20 cm to a depth of 15 cm) of soil were taken from 12 locations along a 'W' line across a field of 600 m^2^ size. After collection, the soil was air dried to ~30% gravimetric moisture content, sieved to <2 mm and stored at 4°C until preparation of the experiment. The experimental design tightly constrained several factors to study the effects of nutrient concentration and fertiliser application area as previously described.^27^ The soil moisture was adjusted to 85% water filled pore space (WFPS) to promote denitrification conditions, taking the amendment with nutrient solution into account. Before starting the experiment, the soil was preincubated to avoid the pulse of respiration associated with wetting dry soils.^31^ For this, the required soil was spread to 3–5 cm thickness. Then, while being mixed continuously, the soil was primed by spraying it with water containing 25 kg N ha^−1^ of potassium nitrate (KNO_3_), which is a typical yearly rate of N deposition through rainfall in the UK.^32^, ^33^ The soil was then left for 3 days at room temperature before being packed into cores and the incubation being started. This was done to promote the growth of denitrifying organisms and prevent a long lag‐phase, therefore reducing the length of the experiment.

**Figure 1:**
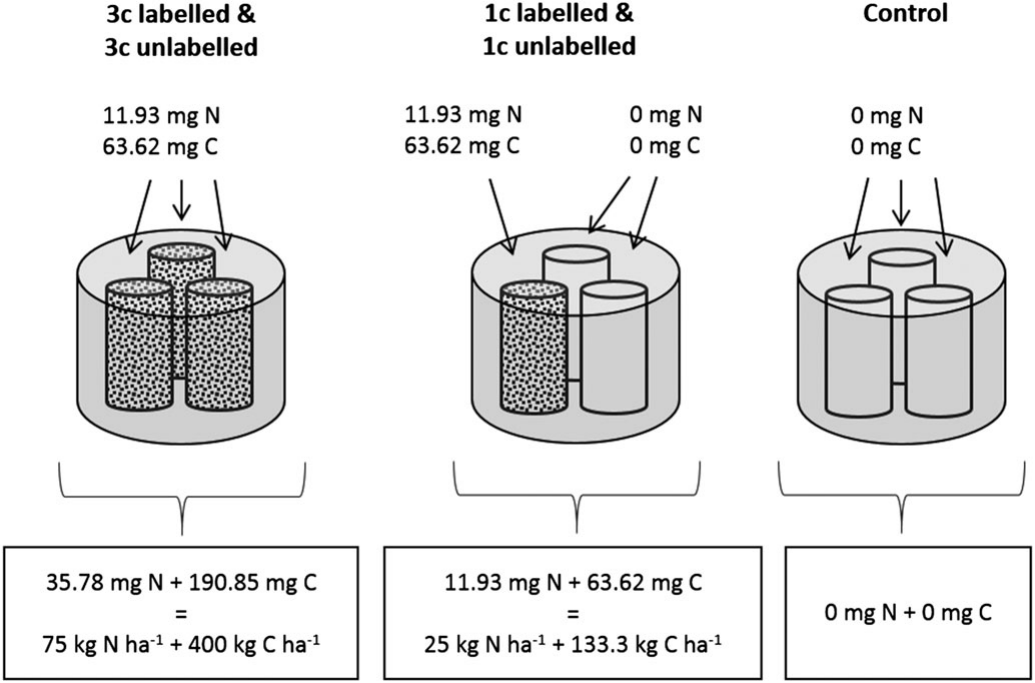
Schematic showing the N and C application rates and amounts of added N and C with the different treatments. Top values are amounts of N and C in mg added per core; bottom values are amounts of N and C in mg added to the whole vessel and the rate this equates to in kg ha^‐1^ per vessel: 3c = nutrients applied to all three cores; 1c = nutrients applied to one core; Control = no nutrient application to any core. Each small core contained 95.3 g dry soil

The incubation experiment was carried out in a specialised gas‐flow‐soil‐core incubation system (DENItrification System (DENIS)^3^) in which environmental conditions can be tightly controlled. The DENIS simultaneously incubates 12 vessels containing 3 soil cores each (Figure 1). The cores were packed to a bulk density of 0.8 g cm^−3^ to a height of 75 mm into plastic sleeves of 45 mm diameter. The vessels were purged to exclude atmospheric N_2_ from the soil and headspace with a He/O_2_ mixture (80:20) as described by Loick et al.^27^ The vessels were kept at 20°C during flushing as well as for the 12‐day incubation period after amendment application. The experiment was set up to investigate the effect of a heterogeneous distribution of N and C on gaseous emissions from denitrification, by applying the same amount of N and C to each of the three cores within a vessel (100% of total surface area, treatment 3c) or to one of the three cores (33% of total surface area, treatment 1c) (Figure 1). The treatments were physically separated into different cores to remove subsurface lateral dispersion effects and to control the mass transfer coefficient at the surface (see Loick et al^27^ for further description).

The experiment was carried out with four replicate vessels per treatment (Figure 1): treatment 1c = one of the three cores inside a vessel was amended with KNO_3_ and glucose; treatment 3c = all three of the cores inside a vessel were amended with KNO_3_ and glucose; Control = only water was applied to each of the three cores. Within each of the treatments 1c and 3c treatments two of the four vessels received ^15^N‐labelled KNO_3_ (5 at%). The experiment was carried out twice, resulting in four labelled and four unlabelled replicates per treatment. Considering the total surface area of the vessel (sum of the areas of the three cores in a vessel), N was applied at a rate of 75 kg N ha^−1^ and C as glucose at 400 kg C ha^−1^ for treatment 3c where N and C were diluted in 15 mL water and 5 mL of that solution was added to each of the three cores inside one vessel. For treatment 1c, N was applied at a rate of 25 kg N ha^−1^ and C as glucose at 133.3 kg C ha^−1^, being applied in solution with 5 mL water to one of the three cores, while the other two cores each received 5 mL water only. The amendment was applied to each of the three cores via a syringe through a sealed port on the lid of the incubation vessel.

### 2.2 Gas analyses and data management

The gas emissions were measured every 10 min consecutively in vessels 1 to 12, resulting in bi‐hourly measurements for each vessel. The fluxes of N_2_O, CO_2_ and N_2_ were quantified by gas chromatography using an electron capture detector (ECD) for N_2_O, and a helium ionization detector (HID) for CO_2_ and N_2_, respectively, while the NO concentrations were determined by chemiluminescence, as described by Loick et al.^27^ The flow rates through the vessel were measured daily and used to correct all gas concentrations and convert them into flux units (kg N or C ha^−1^ d^−1^). The CO_2_ fluxes showed constant emissions of 0.67 kg C ha^−1^ h^−1^ before and after the peak in all vessels, which we consider to be a baseline flux. In order to show emissions attributed to amendment application only, the CO_2_ fluxes in all the treated vessels were adjusted by subtracting this baseline. The initial emission rates for each gas and vessel were determined from the beginning of each peak until the increase in concentrations slowed down, as previously described by Loick et al.^27^

### 2.3 Analysis of the isotopocules of N_2_O

Gas samples for isotopocule analysis of the emitted N_2_O were taken 4 h after amendment application and then daily from unlabelled and control treatments. Samples were collected in two 115‐mL septum‐capped serum bottles, which were connected in line to the vent of each vessel. The isotopocule signatures of N_2_O, i.e. δ^18^O (δ^18^O‐N_2_O) values, average δ^15^N (δ^15^N^bulk^‐N_2_O) values and δ^15^N values from the central N‐position (δ^15^N^α^), were determined by isotope ratio mass spectrometry.^7^ The ^15^N site preference (SP) was obtained as SP = 2 * (δ^15^N^α^ – δ^15^N^bulk^‐N_2_O). The isotopocule ratios of a sample were expressed as ‰ deviation from the ^15^N/^14^N and ^18^O/^16^O ratios of the reference standard materials, atmospheric N_2_ and standard mean ocean water, respectively, as described by Bergstermann et al.^25^

### 2.4 Isotopic analysis of N_2_O in ^15^N‐labelled treatments

Gas samples for ^15^N analysis were taken just before (0 h) and 4 h after amendment application and then daily for the first week, followed by a final sampling at day 11. The sampling dates were chosen to cover changes in isotopic ratios during the main period of NO and N_2_O fluxes, and after the emissions returned to background levels. Samples were taken from the outlet line of each vessel using 12‐mL exetainers (Labco, Lampeter, UK) which had previously been flushed with He and evacuated. The ^15^N‐enrichment of N_2_O was determined using a TG2 trace gas analyser (Sercon, Crewe, UK) and an autosampler (Gilson, Dunstable, UK), interfaced to a Sercon 20–22 isotope ratio mass spectrometer. Standard solutions of 6.6 and 2.9 at% ammonium sulfate ((NH_4_)_2_SO_4_) were prepared and used to generate samples of 6.6 and 2.9 at% N_2_O^34^ which were used as reference and quality control standards. The ^15^N content of the N_2_O was calculated as described by Loick et al^27^ to determine how much of the measured N_2_O derived from the NO_3_^−^ amendment rather than the native soil N.

### 2.5 Soil analyses

The moisture contents and NH_4_^+^ and NO_3_^−^ concentrations were determined in soil samples taken at the beginning and end of the incubation. At the end of the soil incubation time, each core was divided in half to separate the top section from the bottom section. The WFPS was calculated from the soil moisture contents by drying a subsample (50 g) at 105°C overnight. The soil NH_4_^+^‐N and NO_3_^−^‐N were measured by automated colorimetry from 2 M KCl soil extracts using a SANPLUS analyser (Skalar Analytical B.V., Breda, The Netherlands).^35^

### 2.6 Model refinement

A comparison of modelled and measured data for the previously used Rayleigh model^26^ and the Rayleigh model adapted to the N_2_O isotopocule data (determined in this study) was applied to account for isotope effects associated with N_2_O reduction, taking emissions from two distinct soil pools (NO_3_^−^ added with the amendment = pool 1; native soil NO_3_^−^ = pool 2) into account. The previously used Rayleigh model^26^ assumes an exponential increase in the N_2_O originating from pool 2 after amendment application until nitrate in pool 1 is exhausted. However, this exponential increase was only an assumption and not experimentally confirmed. Hence, we used the ^15^N‐labelled treatments to determine the equation that best describes the mixing dynamics of the two NO_3_^−^ pools. The Rayleigh model was then run with the isotopocule data from the unlabelled treatments, but using the equation determined before using the ^15^N‐labelled treatments. In this study, the volume reached by the amendment (volume of pool 1) was assumed to be 33% and 100% in treatments 1c and 3c, respectively. For modelling, we applied the equations described in Lewicka‐Szczebak et al.^26^ Briefly, the isotopic signature of the product, N_2_O and the isotopic signature of the remaining substrate, NO_3_^−^, was calculated according to Equation 1:

(1) $$\frac{\mathrm{\delta S}-1000}{\mathrm{\delta S}0-1000}=f^{\frac{\eta P-S}{1000}}$$

where δ_S_ is the isotopic signature of the remaining NO_3_^−^ (δ^15^N_NO3‐r_); δ_S0_ the isotopic signature of the initial NO_3_^−^ (δ^15^N_NO3‐i_), i.e., fertiliser or soil NO_3_^‐l^; and η_P‐S_ the Net Isotope Effect (NIE) between product and substrate.

In this study, we determined the δ^15^N value of the applied fertiliser whereas that of soil NO_3_^−^was adapted from the literature^26^

δ^15^N_soil NO3_‐ = 10‰.

*f*, the fraction of unreduced NO_3_^−^N, was determined by subtracting the initial NO_3_^−^ concentration and the cumulative N loss as denitrification products (N_2_ + N_2_O) for each time step of the process:

(2) $$f=\left(N_{\mathrm{NO}3-i}-N_{N2+N2O}\right)/N_{\mathrm{NO}3-r}$$

It was assumed that the NO and NO_2_^−^ pools were negligible in the overall N balance, as these represent very reactive intermediate products undergoing fast further reduction. η_P‐S_ represents the Net Isotope Effect (NIE) of N_2_O production referred to as η_N2O‐NO3_. The δ^15^N_N2O‐p_ (instantaneously produced N_2_O) value was calculated according to Equation 3:

(3) $$\delta^{15}N_{N2O-p}≅\delta^{15}N_{\mathrm{NO}3-r}+\eta^{15}N_{N2O-\text{NO3}}$$

The isotopic signature of the reduced N_2_O was calculated according to Equation 1, where δ_S_ is the isotopic signature of the remaining unreduced N_2_O (δN_2_O‐r); δ_S0_ the isotopic signature of the instantaneously produced N_2_O (δN_2_O‐p); *f* the fraction of unreduced N_2_O, calculated based on direct measurements of the N_2_O and N_2_ flux, i.e., the product ratio (N_2_O/(N_2_O + N_2_)); and η_P‐S_ is the NIE of N_2_O reduction referred to as η_N2‐N2O_.

### 2.7 Statistical analysis

Data were analysed to determine normality (Kolmogorov–Smirnov test) and equality of variance (Levene test) conditions. To fulfil these assumptions, the data were log‐transformed before analysis, if needed. Statistical analysis was performed using GenStat 16th edition (VSN International Ltd, Hemel Hempstead, UK). Cumulative emissions were calculated after linear interpolation of the area between sampling points. Differences in total emissions between treatments for each gas measured were assessed by analysis of variance (ANOVA) at *p* <0.01.

## 3 RESULTS

### 3.1 Fluxes and cumulative gas emissions

**Figure 2:**
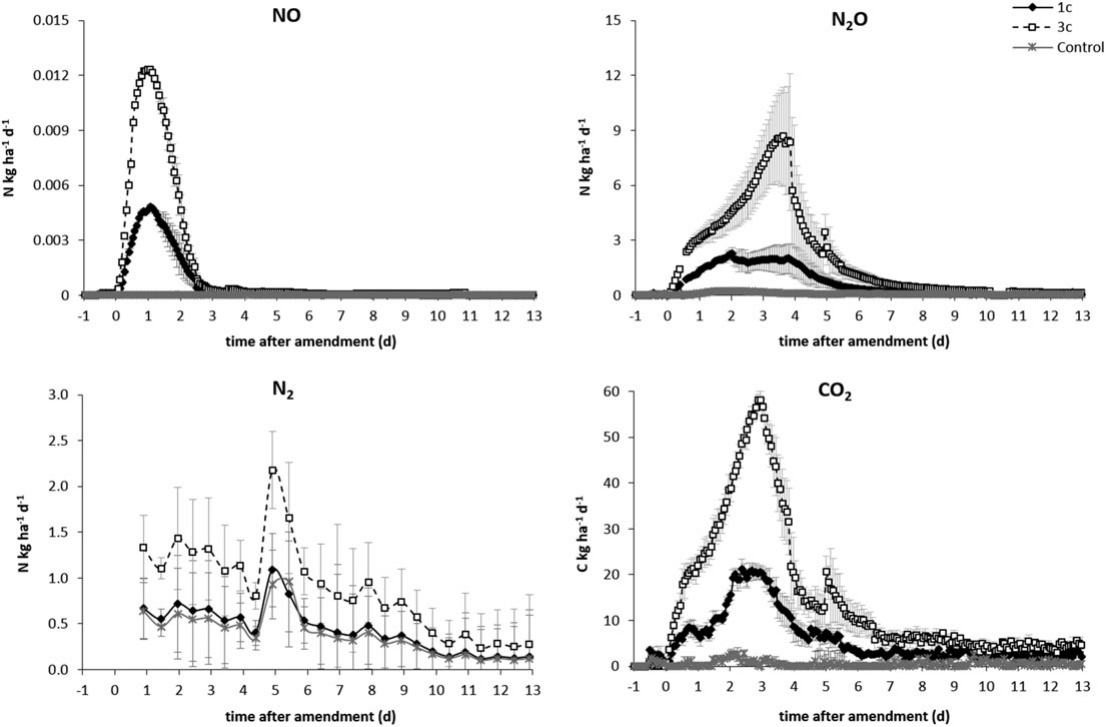
Average fluxes of NO, N_2_O, N_2_ and CO_2_ for the different treatments (*n* = 8). In treatment 1c one of the three cores inside a vessel was amended with KNO_3_ and glucose (the other two received water); in treatment 3c, all three of the cores inside a vessel were amended with KNO_3_ and glucose (each core received the same N and C rate as treatment 1c); in the Control treatment, only water was applied to each of the three cores

The fluxes and cumulative emissions of NO, N_2_O, N_2_ as kg N ha^−1^ and CO_2_ are shown in Figure 2 and Table 1, respectively. The NO emissions from treatments 1c and 3c increased immediately after amendment application with a peak lasting just over 2 days and a maximum on day 1 (Figure 2) The mean cumulative NO emissions from treatment 3c (same shape) was about 2.3 times greater over the time of the incubation than that from treatment 1c (Table 1). Emissions of NO from the Control treatment were negligible.

**Table 1:** Cumulative emissions of NO, N_2_O, N_2_ as kg N ha^−1^ and CO_2_ as kg C ha^−1^. Values were determined in the period between the start and end of the emission peak: NO day 0–4, N_2_O day 0–10, N_2_ day 4.5 to 9.5, CO_2_ day 0–10 after amendment application. Different letters indicate a significant difference between treatments for each measured gas (*n* = 8 for 1c and 3c, *n* = 4 for control; p <0.05). Standard errors of the mean are included

Gas	1c	3c	Control
NO	0.0079 ± 0.0005^B^	0.0183 ± 0.0021^A^	0.0018 ± 0.0003^C^
N_2_O	6.73 ± 1.37^B^	19.49 ± 5.04^A^	1.14 ± 0.13^C^
N_2_	2.88 ± 0.56^B^	5.91 ± 2.25^A^	3.02 ± 0.93^B^
CO_2_	192.23 ± 3.65^B^	313.66 ± 10.07^A^	122.41 ± 6.73^C^
Total N	9.46 ± 1.01^B^	26.12 ± 6.59^A^	4.28 ± 0.89^B^

Similarly to the observed NO emissions, the N_2_O emissions increased immediately after amendment application (Figure 2). The emissions from treatment 3c peaked 3.5 days after the amendment was applied, before decreasing again. The maximum N_2_O emission was larger for treatment 3c than for treatment 1c. In treatment 1c, however, there was a plateau in N_2_O emissions from about day 2 to day 4 before showing the same decrease as treatment 3c. The cumulative emissions of N_2_O (Table 1) were 2.9 times greater from treatment 3c than from treatment1c. The Control treatment only showed very small N_2_O emissions from 1 to 2.5 days after water addition.

The N_2_ fluxes increased after amendment application in treatments 1c and 3c and water addition in the Control treatment (Figure 2). Slightly higher N_2_ fluxes were measured in treatment 3c than in treatment 1c and the Control treatment, showing a peak after 2 days in treatment 3c (Figure 2). In contrast to the NO and N_2_O emissions, the N_2_ cumulative emissions were similar for treatment 1c and the Control treatment, whereas significant higher N_2_ cumulative emissions were measured in treatment 3c (Table 1).

The total denitrification was calculated as the sum of all the N emitted (Table 1) and was significantly higher in treatment 3c than in treatment 1c (2.8‐fold) and the Control (6.1‐fold) treatment.

The CO_2_ fluxes showed similar trends to the N_2_O fluxes. In treatments 1c and 3c, the CO_2_ emissions increased immediately after amendment application (Figure 2) and peaked after about 3 days in both treatments. The cumulative emissions of CO_2_ (Table 1) were 1.6 and 2.6 times greater from treatment 3c than from treatment 1c and the Control treatment, respectively. CO_2_ emissions above background levels were negligible for the Control treatment.

### 3.2 Soil mineral N

The results of the soil analysis at the end of the incubation are given in Table 2. The NO_3_^−^ concentrations were significantly different between the top and the bottom half of the cores for the amended treatments but no significant difference was detected within the Control treatment. The results, if considering the whole vessel, did, however, show that there was a significant difference in the NO_3_^−^ concentrations between treatments 1c and 3c in the top layer (*p* <0.05). Both amended treatments showed significantly higher NO_3_^−^ concentrations than those in the Control treatment.

**Table 2:** Soil characteristics at the end of the experiment. Total amounts measured for nitrate (NO_3_^−^) and ammonium (NH_4_^+^). ‘1c’ = average values for 12 cores (4 amended with 75 kg N ha^−1^, 8 unamended) from vessels of treatment 1c; ‘3c’ = average values for 12 cores (12 amended with 75 kg N ha^−1^) of treatment 3c; ‘control’ = average of 12 cores from the control treatment only receiving water. WFPS values are an average over all three treatments (average values for 36 cores). Different letters indicate a significant difference between treatments for each layer (top or bottom); * indicates significant difference between the top and bottom layer within a single grouping. (*n* = 10 for ‘1c’ and ‘3c’, *n* = 4 for ‘control’), *p* < 0.05). Standard errors are included. NO_3_^−^‐N (mg g^−1^ dry soil) values were 4.6 10^−2^ ± 2.0 10^−4^ and 9.8 10^−3^ ± 4.0 10^−4^ before and after priming, respectively, before amendment application. NH_4_^+^‐N (mg g^−1^ dry soil) amount was 6.0 10^−3^ ± 9.0 10^−6^ before amendment application

Parameter	Layer	1c	3c	Control
NO_3_^−^ (mg N g^−1^ dry soil)	Top	1.44 ± 0.06^B*^	1.68 ± 0.05^A*^	1.23 ± 0.13^B^
	Bottom	1.28 ± 0.04^A*^	1.36 ± 0.04^A*^	1.13 ± 0.03^B^
NH_4_^+^ (mg N g^−1^ dry soil)	Top	0.055 ± 0.002^B*^	0.050 ± 0.001^C*^	0.060 ± 0.001^A*^
	Bottom	0.069 ± 0.004^A*^	0.066 ± 0.003^A*^	0.076 ± 0.005^A*^
WFPS (%)	Top	83.2 ± 0.50^*^		
	Bottom	76.0 ± 0.56^*^		

Regardless of the treatment, the NH_4_^+^ concentrations were lower than the NO_3_^−^ concentrations at the end of the incubation, with significantly higher values in the bottom layer of the core. Both soil NH_4_^+^ and NO_3_^−^ increased in all treatments compared with the initial soil conditions (6.1 and 15 mg N kg dry soil^−1^). The NH_4_^+^ concentrations were only significantly different between treatments in the top layer, in decreasing order: Control >1c > 3c. The soil moisture content was significantly different between the top (83.2 ± 0.50) and the bottom (76.0 ± 0.56) half of the cores at the end of the incubation in all treatments.

### 3.3 ^15^N‐enrichment of N_2_O in the ^15^N‐labelled treatment

**Figure 3:**
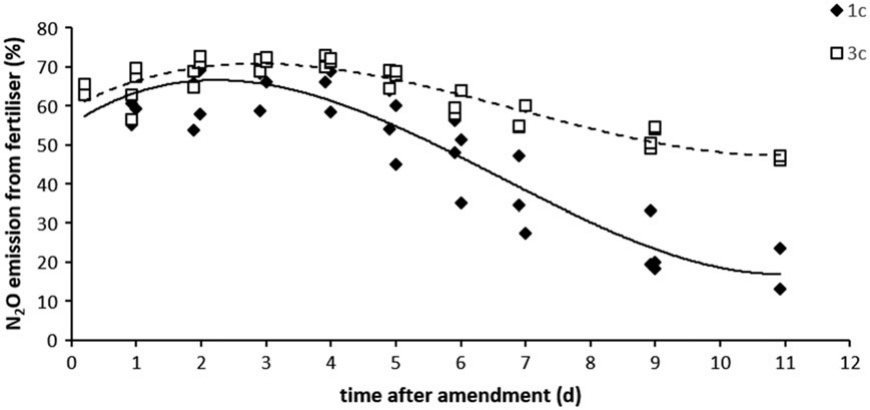
Contribution of applied fertiliser‐N to N_2_O emissions as determined from ^15^N‐enrichment of the emitted N_2_O from those 1c and 3c treatments that had received ^15^N‐labelled KNO_3_ with their amendment

**Figure 4:**
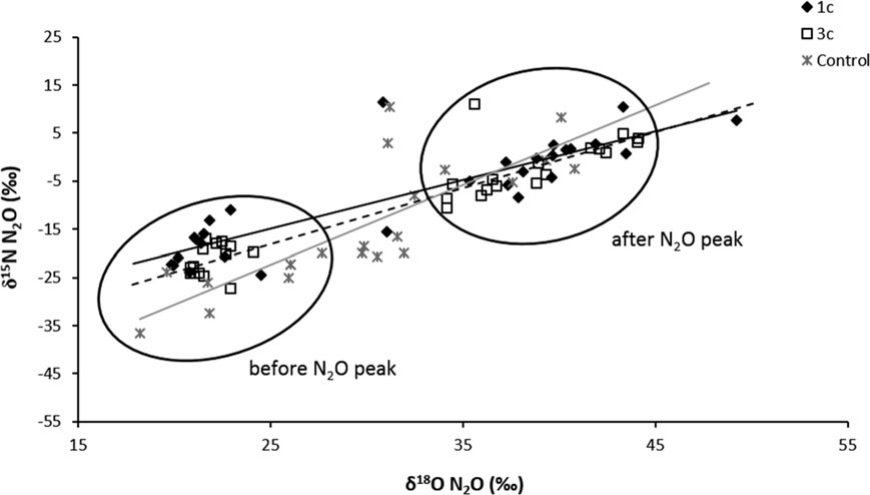
Comparison of δ^15^N bulk and δ^18^O values of soil‐emitted N_2_O from those 1c and 3c treatments that had received unlabelled KNO_3_ with their amendment as well as the Control treatment

The ^15^N‐enrichment of the emitted N_2_O is shown in Figure 3. Regardless of the N treatment, up to day 4 around 70% of the emitted N_2_O was derived from the applied amendment, with a constant decrease thereafter (Figure 3). After 4 days, when N_2_O emissions decrease while the N_2_ fluxes increase (Figure 4), which indicates that N_2_O reduction dominates over N_2_O production, the enrichment in ^15^N of the N_2_O decreases. This decrease is faster in treatment 1c than in treatment 3c, reaching a final contribution of fertiliser N to N_2_O emissions of around 20% and 50%, respectively, by day 11.

### 3.4 Isotopic signature of N_2_O in the non‐labelled treatments

#### 3.4.1 δ^15^N^bulk^ values of N_2_O

The δ^15^N^bulk^‐N_2_O values were not significantly different between the N‐amended treatments during the first 4 days, and increased from an initial value of about −23.4‰ in both treatments to −1.1‰ and − 5.5‰ in treatments 1c and 3c, respectively (Table 3). After 4 days, the δ^15^N^bulk^‐N_2_O values remained relatively constant in treatment 3c, in the range of −1.2 to 1.7‰, until the end of the incubation. In contrast, in treatment 1c the δ^15^N^bulk^‐N_2_O values increased until day 6 (10.4‰) and declined by day 9 (−4.2‰), peaking again on day 11 (51.8‰). Immediately after water addition, the δ^15^N^bulk^‐N_2_O value of the Control treatment was −23.8‰ and it peaked on day 6 (10.4‰) to decrease afterwards until −20.7‰ on day 11 (Table 3).

**Table 3:** Measured isotopic ratios of emitted N_2_O, as δ^18^O, δ^15^N^bulk^ and site preference (SP), in those 1c and 3c treatments that received unlabelled KNO_3_ with their amendment as well as the control treatment over the time of the incubation

Days after treatment	δ^18^O values (‰)			δ^15^N^bulk^ values (‰)			SP (‰)		
	1c	3c	Control	1c	3c	Control	1c	3c	Control
0	25.6	24.0	39.7	−23.4	−23.3	−23.8	−1.6	−4.9	22.4
2	21.4	21.7	18.9	−18.0	−16.9	−26.0	−6.0	−5.7	−4.1
4	37.3	38.9	30.1	−1.1	−5.5	−8.1	−6.3	−5.5	−3.7
6	43.3	41.7	31.1	10.4	−1.2	10.4	3.6	1.8	3.9
9	39.6	42.4	31.9	−4.2	1.0	−19.8	7.0	3.1	6.4
11	42.1	42.1	37.9	51.8	1.7	−20.7	9.4	4.3	22.9

#### 3.4.2 ^15^N site preference of N_2_O

The ^15^N site preference of N_2_O (SP‐N_2_O) of both N‐amended treatments decreased slightly for the first 4 days and gradually increased thereafter until the end of the incubation, showing only small differences between them (Table 3). Overall, the SP N_2_O values increased from an initial value in the range of −1.6 and −4.9‰ to a maximum of approximately 9.4‰ and 4.3‰ in treatments 1c and 3c, respectively (day 11 after application). The SP N_2_O from the Control treatment increased after the application of water up to 22.5‰ and declined to −4.1‰ by day 2, increasing gradually until the end of the incubation to reach a final value of 22.9‰ (Table 3). The δ^15^N^α^ and δ^15^N^β^ values followed a similar trend to the δ^15^N^bulk^ values with small differences between the isotope ratios, and generally δ^15^N^α^ > δ^15^N^β^ (data not shown).

#### 3.4.3 δ^18^O values of N_2_O

Similar to the N_2_O SP, the δ^18^O values of N_2_O showed small differences in the temporal pattern between treatments 1c and 3c (Table 3). Overall, the δ^18^O values of the N_2_O in both N‐amended treatments increased continuously from an average 29.4‰ to 40.4‰ at the end of the incubation. In the Control treatment, the δ^18^O values of N_2_O increased after water application to 39.7‰, followed by a decline to 18.9‰ by day 2. Afterwards, the value gradually increased until the end of the incubation to about 37.6‰ (Table 3).

An X/Y plot of δ^18^O‐N_2_O values against δ^15^N^bulk^‐N_2_O values is presented in Figure 4. Regardless of the treatment, both isotope ratios increased at a ratio of approximately 1:3 during the incubation. A similar behaviour was observed in both N‐amended treatments, which indicated that the ratio of the simultaneous increase in the δ^18^O‐N_2_O and δ^15^N^bulk^‐N_2_O values did not differ between treatments (Figure 4). Moreover, the δ^18^O‐N_2_O and δ^15^N^bulk^‐N_2_O values grouped into two separate clusters depending on whether they were measured from samples taken before or after the N_2_O peak. As expected, a different trajectory in the δ^15^N^bulk^‐N_2_O and δ^18^O‐N_2_O values was observed in the Control treatment over the experimental period.

**Figure 5:**
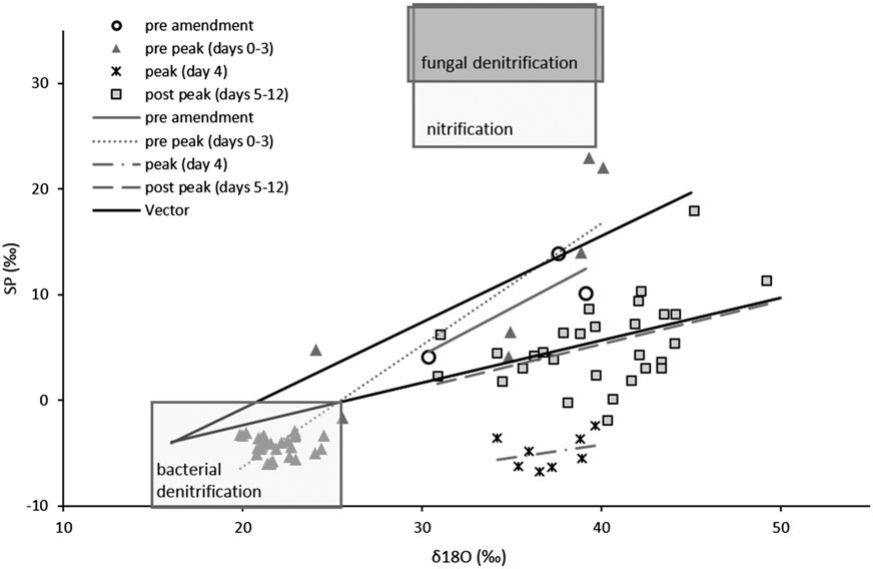
SP vs δ^18^O values from all vessels that had received unlabelled amendment, grouped for four time periods depending on the appearance of the peak in N_2_O emissions (circles = pre‐amendment; triangles = after amendment application, but before the N_2_O peak (days 0–3); crosses = during the N_2_O peak (day 4); squares = post N_2_O peak (days 5‐12), all with associated trendlines (see legend)). The solid black lines are reduction lines after Lewicka‐Szczebak et al^18^ representing minimum and maximum routes of isotopocule values with increasing N_2_O reduction to N_2_. Endmember areas for fungal denitrification, nitrification and bacterial denitrification are from Lewicka‐Szczebak et al^18^

The X/Y plot of δ^18^O‐N_2_O values against SP in Figure 5 shows the “map” for the values of δ^18^O and SP from all unlabelled treatments. Reduction lines (vectors) represent minimum and maximum routes of isotopocule values with increasing N_2_O reduction to N_2_ based on the reported range in the ratio between the isotope fractionation factors of N_2_O reduction for SP and the δ^18^O values.^18^ Most of the values measured after amendment application, but before the N_2_O peak, are below the lower reduction line, but within the area indicating bacterial denitrification. During the N_2_O peak the samples show increased δ^18^O values followed by an increased SP after the peak.

#### 3.4.4 Modelling ^15^N‐enrichment of N_2_O

Measurements of ^15^N‐enrichment using the ^15^N‐labelled treatments 1c and 3c (Figure 3) derived in the polynomial Equations 4 and 5, respectively, were:

(4) $$f\left(x\right)=0.14{\text{8x}}^{3}–2.9435x^{2}+10.89\text{2x}+55.28;R^{2}=0.8532$$

(5) $$f\left(x\right)=0.09{\text{2x}}^{3}–1.8938x^{2}+8.5897x+59.56;R^{2}=0.8514$$

where f(X) is the contribution of fertiliser N to N_2_O in % and x is the time after amendment (d).

**Figure 6:**
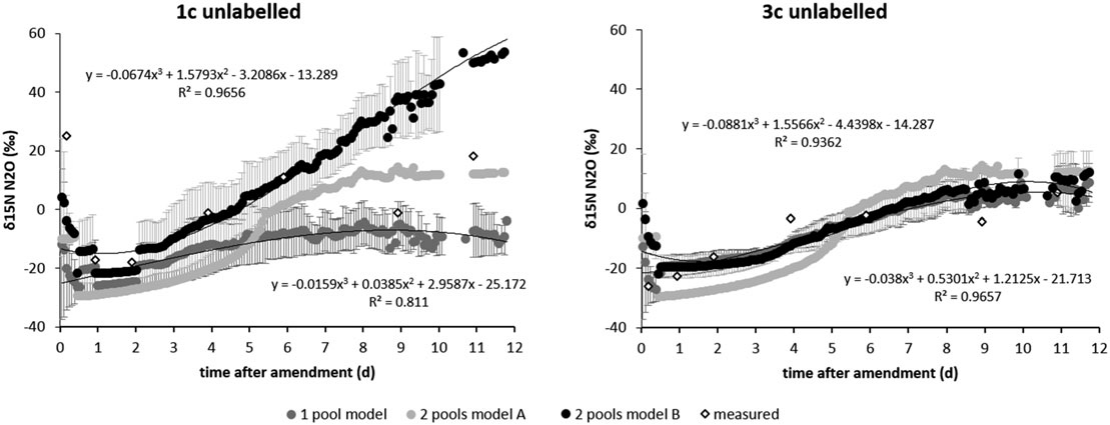
Comparison of modelled and measured data for the previously used Rayleigh model (model A) and the Rayleigh model adapted according to ^15^N data (model B) for the two treatments 1c (left) and 3c (right) assuming one‐pool emission (only from fertiliser) and two‐pool emission (from fertiliser and soil nitrate). Equations relate to the adapted two‐pool model B (top equation) and the one‐pool model (bottom equation)

The Rayleigh model fit adapted to ^15^N data for the unlabelled treatments 1c and 3c was evaluated in all vessels, assuming one‐pool and two‐pool emissions. Only two vessels per treatment (*n* = 4) showed a good polynomial fit (R^2^ >0.89) of the modelled data to the measured data and an average of them is shown in Figure 6. The equations and R^2^ values of all the vessels for each N pool are shown in Table S1 (supporting information). The Rayleigh model applied to the δ^15^N^bulk^‐N_2_O data showed poor agreement with the measurements using the original model for treatment 1c, with the two‐pool model giving better results when using the polynomial equation determined above (Figure 6). In contrast, for treatment 3c little difference was observed between the modelling approaches (Figure 6).

## 4 DISCUSSION

### 4.1 Soil data and gaseous emissions

Our findings are in agreement with those of Wang et al^36^ and Loick et al^27^ who found that the emissions of NO, N_2_O and CO_2_ are related to the amounts of applied NO_3_^−^ and C, NO_3_^−^ and C thereby being the limiting factors for denitrification activity, rather than the soil area and volume and associated microbial population that receives the amendment. Although the total emissions were not similar, the peaks of N_2_O, NO and CO_2_ fluxes were concurrent in treatments 1c and 3c. Moreover, the amendment solution was spread over all three cores in treatment 3c which could have potentially supported a three times larger microbial community with the nutrients than treatment 1c. Loick et al^27^ found a delay in the N_2_O emission peak when only one of three cores inside a vessel was amended with the full amount of nutrients, compared with an equal distribution of the treatment into three cores (so each core received 1/3 of the nutrients). In our case, in treatments 1c and 3c all individual cores (one in 1c and three in 3c) received the same amount of nutrients and the response time was similar, showing that denitrifiers transformed the NO_3_^−^ added to N_2_O for the same time period in both treatments, regardless of the soil area/volume amended. Although the cumulative emissions of N_2_ were higher in treatment 3c, the fluxes were lower than the N_2_O fluxes in all treatments. It has been demonstrated that many denitrifiers lack one or more of the denitrification enzymes involved in all reduction steps from NO_3_^−^ to N_2_,^37^ particularly N_2_O reductase (NosZ) the enzyme reducing N_2_O to N_2_. In addition, the last step in denitrification is also the least energetically favourable.^38^ Therefore, denitrifiers would preferentially reduce NO_3_^−^ to N_2_O rather than N_2_O to N_2_. We hypothesised that these reasons explain the accumulation of N_2_O over N_2._ 27, 39

### 4.2 Isotope analysis of N_2_O from ^15^N‐labelled treatments

The ^15^N signature of N_2_O was used to determine the contribution of the native soil NO_3_^−^ or the NO_3_^−^ added with the amendment to the N_2_O emissions (Figure 3). While in treatment 3c N_2_O emissions were mainly from the added NO_3_^−^ (pool 1) throughout the whole experimental period, in treatment 1c, a low ^15^N enrichment of the measured N_2_O was observed after 5 days, indicating that after this time most of the emitted N_2_O was from the native soil NO_3_^−^ (pool 2). This can be explained due to NO_3_^−^ limitation in the soil treated in treatment 1c after the N_2_O peak. Because only one‐third of the soil/microbial community received nutrient amendment, N_2_O emissions were low in treatment 1c and those from the non‐amended cores are likely to mask the effect of the amendment on N_2_O production.^27^ Moreover, after 11 days, N_2_O production in treatment 3c still came from the NO_3_^−^ added.

### 4.3 Analysis of isotopocules of N_2_O

#### 4.3.1 δ^15^N^bulk^‐N_2_O values

The increase in δ^15^N^bulk^‐N_2_O values until day 4 in both treatments 1c and 3c is probably a consequence of the ^15^N‐enrichment during ongoing NO_3_^−^ reduction of the added NO_3_^−^.^25^ From day 4 onwards the δ^15^N^bulk^‐N_2_O values increased in treatment 1c, indicating enrichment in ^15^N from a different pool of NO_3_^−^. The ^15^N‐enrichment of N_2_O in the ^15^N‐labelled treatment 3c showed that some of the N_2_O (30 to 50%) came from soil‐derived NO_3_^−^. This suggests that pool 1 dominated initially (while the unlabelled treatment showed an increase in δ^15^N^bulk^‐N_2_O values) whereas, when the relative contribution of soil‐NO_3_^−^ increased (which can be seen by lowering of N_2_O emission from fertiliser), the δ^15^N^bulk^ values did not increase further, due to the increasing contribution from pool 2 masking any increases in δ^15^N^bulk^ values from pool 1. In treatment 1c, however, changes in the ^15^N‐enrichment of the N_2_O could be related to the influence of two N‐pools; one core receiving amendment (soil N + added N) and two cores with only soil N with different denitrification dynamics where the fraction of N_2_O varied over time. The observed dynamics are in line with earlier observations during incubation of NO_3_^−^/glucose‐amended soil cores^25^, ^26^ where the initial increase in δ^15^N^bulk^‐N_2_O values had been explained by the fast exhaustion of NO_3_^−^ and the consequential ^15^N‐enrichment of residual NO_3_^−^ from pool 1 during the earlier phase, followed by declining N_2_O fluxes from pool 1 after its exhaustion. The lowering of δ^15^N^bulk^ values was explained as being from the growing contribution of pool 2 to N_2_O fluxes, since pool 2 was previously less fractionated than pool 1 due to its lower denitrification rate in the absence of glucose. The final increase in δ^15^N^bulk^ values was explained by N_2_O fluxes from pool 2 since its NO_3_^−^ was also progressively reduced and thus fractionated. The latter was verified by modelling of the δ^15^N‐N_2_O values and it is further discussed in section 4.4.

#### 4.3.2 The ^15^N site preference

The SP of the N_2_O is the result of several mechanisms responsible for N_2_O production such as nitrification, bacterial and fungal denitrification.^15^, ^40^, ^41^, ^42^ The range of SP values in this study is in agreement with those from previous studies under denitrifying conditions.^18^, ^25^, ^43^ Moreover, it is known that reduction of N_2_O to N_2_ causes ^15^N accumulation on the central N‐position of the N_2_O because of the cleavage of NO bonds during this process.^15^, ^40^ In fact, we observed a N_2_ peak after 5 days, in both treatments 1c and 3c, with higher SP values indicating the reduction of N_2_O to N_2_.

In this study, the decrease in ^15^N SP values of N_2_O before the N_2_O peak followed by an increase suggests that the site‐specific ^15^N fractionation factor of the reduction of NO_3_^−^ to N_2_O was not constant in treatments 1c and 3c. At the end of the experiment, the maximum SP value was reached, coinciding with minimum fluxes of N_2_O and the lowest N_2_O/(N_2_ + N_2_O) ratio, suggesting an increase in the extent of the N_2_O reduction.^25^ Regardless of the amounts of N and total area amended, the variation in the SP N_2_O between treatments was relatively small. This agrees with earlier studies^12^, ^25^, ^43^ that explained the decline in SP values as resulting from the initiation of anaerobic conditions after inducing this process by flushing with N_2_ or with a decreasing contribution from fungal denitrification. It is possible that some N_2_O emission resulted from nitrification although the soil moisture was adjusted to favour denitrification.^7^

#### 4.3.3 The δ^18^O signatures

The values of δ^18^O‐N_2_O are determined by NO_3_^−^, O_2_ and soil H_2_O incorporation and reduction effects during the production of N_2_O resulting in ^18^O‐depleted or ‐enriched N_2_O, respectively, since the ^18^O–N bond is more stable and ^16^O is removed more easily from NO_3_^−^.^41^, ^43^ It is known that oxygen can be incorporated from H_2_O to N_2_O during denitrification to constitute more than 60% of the O in the N_2_O produced‐.^44^, ^45^ During the first four days of the incubation, the δ^18^O‐N_2_O values increased indicating an independence of the δ^18^O‐N_2_O values from the δ^18^O‐NO_3_ values during the production of N_2_O that can be attributed to a lower O‐exchange with water.^12^ Our results are in agreement with those reported by Meijide et al^43^ and Bergstermann et al^25^ showing stabilisation of δ^18^O‐N_2_O values after the N_2_O peak. However, in contrast to Meijide et al^43^ we did not observe an increase in δ^18^O‐N_2_O values linked to an increase of N_2_ fluxes.

In this study, different patterns of δ^15^N^bulk^ vs δ^18^O values (Figure 4 showing two clusters before and after the N_2_O peak as well as differently sloped lines for the different treatments) suggested the temporal change in denitrification between the different pools before and after the N_2_O peak. Before the N_2_O peak, N_2_O originated from non‐fractionated NO_3_^−^ in pool 1 (NO_3_^−^ added from fertiliser) whereas after the N_2_O peak the main flux might have come from pool 2 (mixture from fertiliser and native NO_3_^−^), which also contained less fractionated NO_3_^−^ initially.^43^ Moreover, the patterns of SP vs δ^18^O values gave further indications on processes contributing to N_2_O fluxes:^18^, ^46^ pre‐peak values cluster mainly in the bacterial endmember area indicating little contribution from other sources and minor reduction in agreement with flux data, whereas post‐peak values (>day 4) cluster around the reduction line, indicating bacterial production with varying reduction to N_2_, where the latter is also confirmed by flux data (Figure 3). Interestingly, the peak values form a distinct cluster below the reduction line with SP values below zero per mil, indicative of bacterial production with minor reduction, but the δ^18^O values are increased by 15 to 20‰ compared with the pre‐flux values. Those data can thus not be explained with the “mapping approach” suggested by Lewicka‐Szczebak et al^18^ which assumes that the δ^18^O value of bacterial N_2_O prior to its reduction is relatively constant due to almost complete O‐exchange with water, implying that a positive shift in the δ^18^O value must be due to N_2_O reduction and associated with increasing SP values. Because the δ^15^N_bulk_ values exhibited a similar upshift until day 4, we assume that this effect is due to an increase in the δ^18^O and δ^15^N values of the NO_3_^−^ precursor resulting from fractionation during intense denitrification in this phase of the experiment (day 4). This would also mean, however, that O‐exchange with water during N_2_O production was incomplete, which has been reported earlier for a dynamic incubation similar to our study.^45^

### 4.4 Isotopocules model

The Rayleigh model^25^, ^26^ was applied to account for the importance of N_2_O emissions from the one‐pool and two‐pools using the δ^15^N^bulk^ values of N_2_O. Until now, this model has been used to simulate the δ^15^N values of N_2_O using process rates and associated fractionation factors, but assumptions had to be made for some of the model parameters due to lack of available data.^25^ In this study, we carried out two incubation experiments in order to parameterise the model. The range of δ^15^N^bulk^ values agrees with other studies that identified denitrification as the main N_2_O‐producing process under similar conditions.^43^ Data from ^15^N‐labelling showed an initial increase in the contribution of pool 1 followed by a decrease (Figure 3), which was sooner and larger in treatment 1c. The comparison of the previously used Rayleigh model^25^, ^26^ and the Rayleigh model adapted in this study according to δ^15^N^bulk^ analysis of N_2_O showed that a two‐pool model was better for interpreting treatment 1c, whereas for treatment 3c little difference between the modelling approaches was observed. This supports the idea that the amendment was mixed with parts of the soil pool, forming one uniform pool initially dominating N_2_O emissions in treatment 3c. In this treatment the δ^15^N^bulk^ levels stabilise after day 6, which indicates that a second pool contributes to emissions. Previous studies^25^, ^26^ assumed that during the N_2_O emission peak, a small but increasing contribution from pool 2 also occurs and its contribution was fitted assuming an exponential increase of pool 2 emission until reaching the emission observed after the extinction of pool 1. Using two different amendment areas, we found that a third‐order polynomial equation based on empirical δ^15^N^bulk^ data improved the fit of the model, especially for treatment 1c.

Although we intended to control the magnitude of pool 1 (33% or 100% of amendment area) in this study, the Rayleigh model fit adapted to the ^15^N‐labelling data showed a good third‐order polynomial fit for only two vessels per treatment. Thus, a better parameterising of the model should be addressed for examination of fractionation factors for various product ratios and reaction rates of pool 2 by future studies.

## 5 CONCLUSIONS

Determining N_2_O emissions from different N‐pools in soil is important for the interpretation of N_2_O isotopocule data. This study shows the potential for understanding the source of N_2_O emissions from different N pools using an improved model for the interpretation of N_2_O isotopocule data. It was indicated that the assumptions regarding the exponential increase in pool 2 activity accepted in previous studies^25^, ^26^ should be replaced with a polynomial increase with dependence on both pool sizes. Our results show the value of parameterising models under controlled laboratory conditions using experimental data but further work is required to apply the findings to other soil types and improve the refinement of model parameters.

## Supporting information

Table S1. Rayleigh model adapted equations according to ^15^N data (model B) for the 1C and 3C treatments assuming 1‐pool emission (only from fertiliser) and 2‐pool emission (mixture from fertiliser and soil nitrate). Only vessels with R^2^ value >0.89 (in bold and underlined)
