# Supplementary material for: Improved isotopic model based on 15N tracing and Rayleigh‐type isotope fractionation for simulating differential sources of N2O emissions in a clay grassland soil
Source: Rapid Commun Mass Spectrom. 2019 Feb 15;33(5):449–60. doi: 10.1002/rcm.8374 (PMC6492082; doi:10.1002/rcm.8374)
Supplement: Supplementary file 1 — Table S1. Rayleigh model adapted equations according to 15N data (model B) for the 1C and 3C treatments assuming 1‐pool emission (only from fertiliser) and 2‐pool emission (mixture from fertiliser and soil nitrate). Only vessels with R2 value >0.89 (in bold and underlined) [file RCM-33-449-s001.docx]

**Supplementary Material**

Table S1. Rayleigh model adapted equations according to ^15^N data (model B) for the 1C and 3C treatments assuming 1-pool emission (only from fertiliser) and 2-pool emission (mixture from fertiliser and soil nitrate). Only vessels with R^2^ value > 0.89 (in bold and underlined) were averaged in Figure 5.

| 1C treatment | | |
| --- | --- | --- |
| Replicate | 1 pool model | 2 pools model B |
| 1 | -0.0269 x^3^ + 0.3116 x^2^ + 0.5187 x -12.584; R^2^ = 0.4513 | -0.0468 x^3^ + 1.4199 x^2^ – 3.9778 x -0.5881; R^2^ = 0.9803 |
| **2** | -0.0181 x^3^ + 0.0989 x^2^ – 2.5514 x -29.453; R^2^ = 0.9755 | -0.1486 x^3^ + 2.7691 x^2^ – 8.2328 x -13.852; R^2^ = 0.9934 |
| **3** | -0.0827 x^3^ + 1.5764 x^2^ – 5.4015 x -12.603; R^2^ = 0.9428 | 0.4896 x^3^ + 2.1841 x^2^ + 0.7549 x -7.5495; R^2^ = 0.9296 |
| 4 | -0.083 x^3^ + 0.9837 x^2^ – 2.5384 x -25.597; R^2^ = 0.3296 | -0.1209 x^3^ + 1.8242 x^2^ – 6.0969 x -18.661; R^2^ = 0.7531 |
| 5 | -0.0337 x^3^ + 0.6445 x^2^ – 2.4516 x -23.049; R^2^ = 0.2604 | -0.0232 x^3^ + 0.2143 x^2^ – 1.3607 x -19.67; R^2^ = 0.7876 |
| 3C treatment | | |
| Replicate | 1 pool model | 2 pools model B |
| **1** | -0.0576 x^3^ + 0.9265 x^2^ – 0.8524 x -16.623; R^2^ = 0.9007 | -0.0951 x^3^ + 1.7023 x^2^ – 4.9337 x -11.687; R^2^ = 0.8998 |
| **2** | -0.0245 x^3^ + 0.267 x^2^ + 2.6586 x -26.278; R^2^ = 0.9806 | -0.09 x^3^ + 1.5996 x^2^ – 4.8014 x -16.171; R^2^ = 0.9591 |
| 3 | -0.0215 x^3^ + 0.2477 x^2^ – 0.3877 x -21.19; R^2^ = 0.225 | 0.0071 x^3^ - 0.0184 x^2^ + 0.2017 x -22.828; R^2^ = 0.1847 |
| 4 | -0.734 x^3^ + 11.073 x^2^ – 27.159 x +18.786; R^2^ = 0.8647 | -0.4859 x^3^ + 8.2491 x^2^ – 26.397 x +10.407; R^2^ = 0.7864 |
